# COVID-19-Associated Acquired Hemophilia A With an Exceptionally High Inhibitor Titer: A Case of Remission and Overwhelming Sepsis

**DOI:** 10.7759/cureus.102600

**Published:** 2026-01-29

**Authors:** Shaivya Pathak

**Affiliations:** 1 Internal Medicine, East Carolina University, Greenville, USA

**Keywords:** acquired hemophilia a, bethesda units, covid-19, emicizumab, factor viii inhibitor, hemorrhagic shock, immunosuppression, rituximab, sars-cov-2

## Abstract

Acquired hemophilia A (AHA) is a rare autoimmune bleeding disorder caused by autoantibodies against factor VIII. COVID-19 infection has emerged as a potential trigger, though reported cases remain limited, with variable inhibitor titers. We report a 70-year-old African American man who developed AHA following documented COVID-19 infection in October 2023, presenting with an exceptionally high factor VIII inhibitor titer of 561 Bethesda Units (BU) that peaked at 677 BU during initial treatment. His course was complicated by migratory spontaneous hematomas and a massive chest wall hematoma leading to hemorrhagic shock and pulseless electrical activity (PEA) cardiac arrest, which he survived. Despite the severity, he achieved complete immunological remission with factor VIII normalization to 343% and inhibitor eradication (0 BU) following treatment with FEIBA, rituximab, cyclophosphamide, and corticosteroids. However, approximately three months after his initial diagnosis, he died from multiorgan failure secondary to overwhelming sepsis (procalcitonin 747.64 ng/mL) in the setting of profound treatment-induced immunosuppression. This case represents one of the highest reported inhibitor titers in COVID-19-associated AHA and demonstrates that complete remission is achievable even in severe cases. Yet this case underscores a critical therapeutic paradox: the aggressive immunosuppression necessary for inhibitor eradication carries a substantial risk of infectious mortality. Emerging therapies such as emicizumab may help mitigate this paradox by allowing reduced-intensity immunosuppression, though cost and off-label status currently limit accessibility.

## Introduction

Acquired hemophilia A (AHA) is a rare autoimmune bleeding disorder characterized by the development of autoantibodies (inhibitors) against coagulation factor VIII, with an estimated incidence of 1-4 cases per million population annually [1]. Unlike congenital hemophilia, AHA typically presents in older adults without prior bleeding history, manifesting as spontaneous soft tissue hematomas, extensive ecchymoses, and mucosal bleeding rather than hemarthrosis [2]. The disorder carries significant morbidity and mortality, with reported mortality rates ranging from 8% to 33%, primarily due to hemorrhagic complications and treatment-related infections [3]. Approximately 50% of AHA cases are idiopathic, while the remainder are associated with autoimmune diseases, malignancy, pregnancy, and medications [2]. Since 2020, COVID-19 infection and vaccination have emerged as potential triggers for AHA, with case reports documenting this association [4-6]. However, the number of reported COVID-19-associated AHA cases remains limited, and the typical inhibitor titers in these cases have ranged from 8 to 318 Bethesda units (BU) [7]. Treatment of AHA requires a dual approach: hemostatic management of acute bleeding with bypassing agents (activated prothrombin complex concentrate [FEIBA] or recombinant factor VIIa) and immunosuppressive therapy to eradicate the inhibitor [1]. First-line immunosuppression typically includes corticosteroids with or without cyclophosphamide, with rituximab reserved for refractory cases or added as first-line therapy in severe disease [8].

We present a case of COVID-19-associated AHA with an exceptionally high inhibitor titer of 561-677 BU, complicated by life-threatening hemorrhage and cardiac arrest, who achieved complete immunological remission but subsequently died from treatment-related immunosuppression. This case highlights both the potential for successful inhibitor eradication in severe disease and the critical balance between effective treatment and infectious complications.

## Case presentation

**Figure 1:**
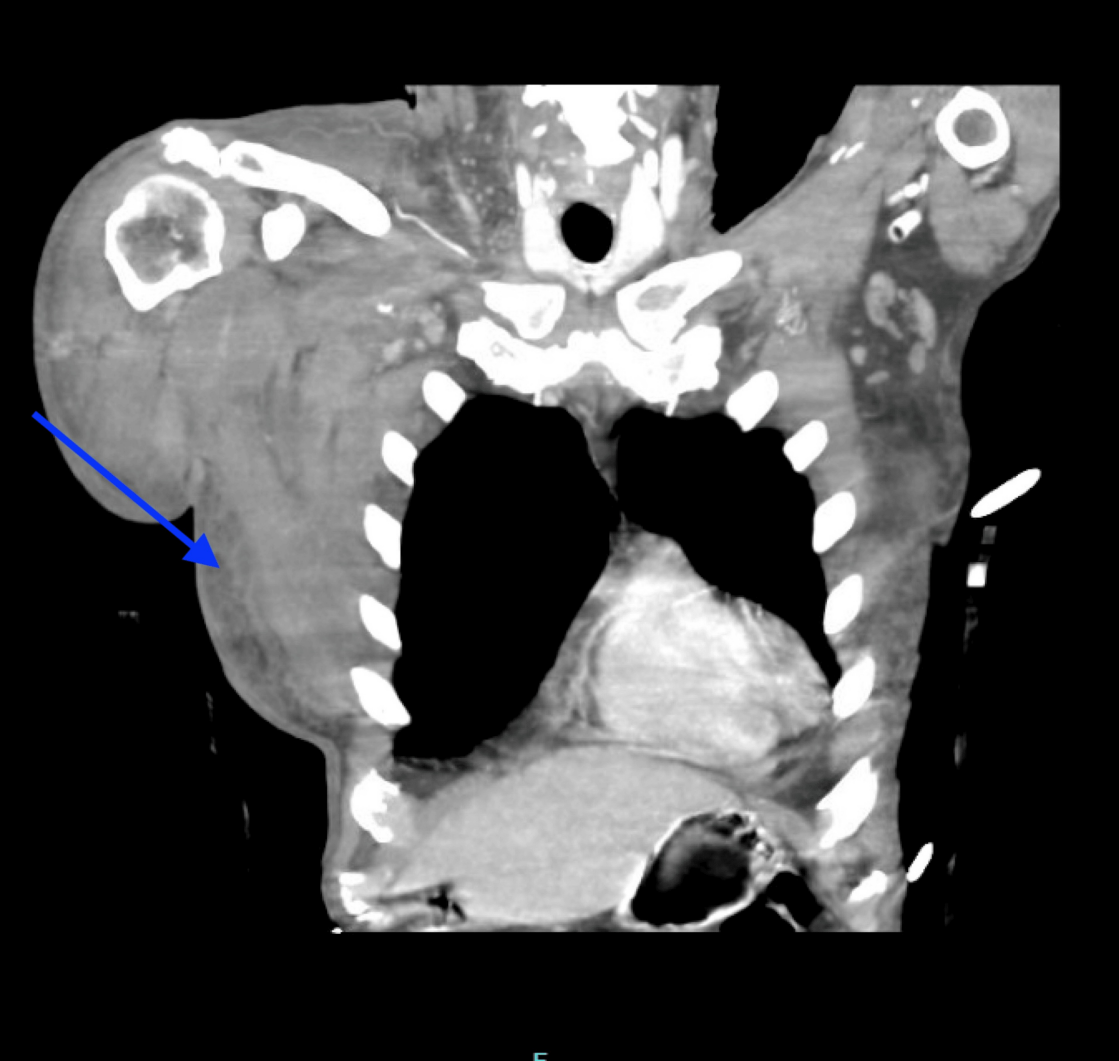
Computed tomography of the chest demonstrating a right chest wall hematoma (blue arrow).

**Figure 2:**
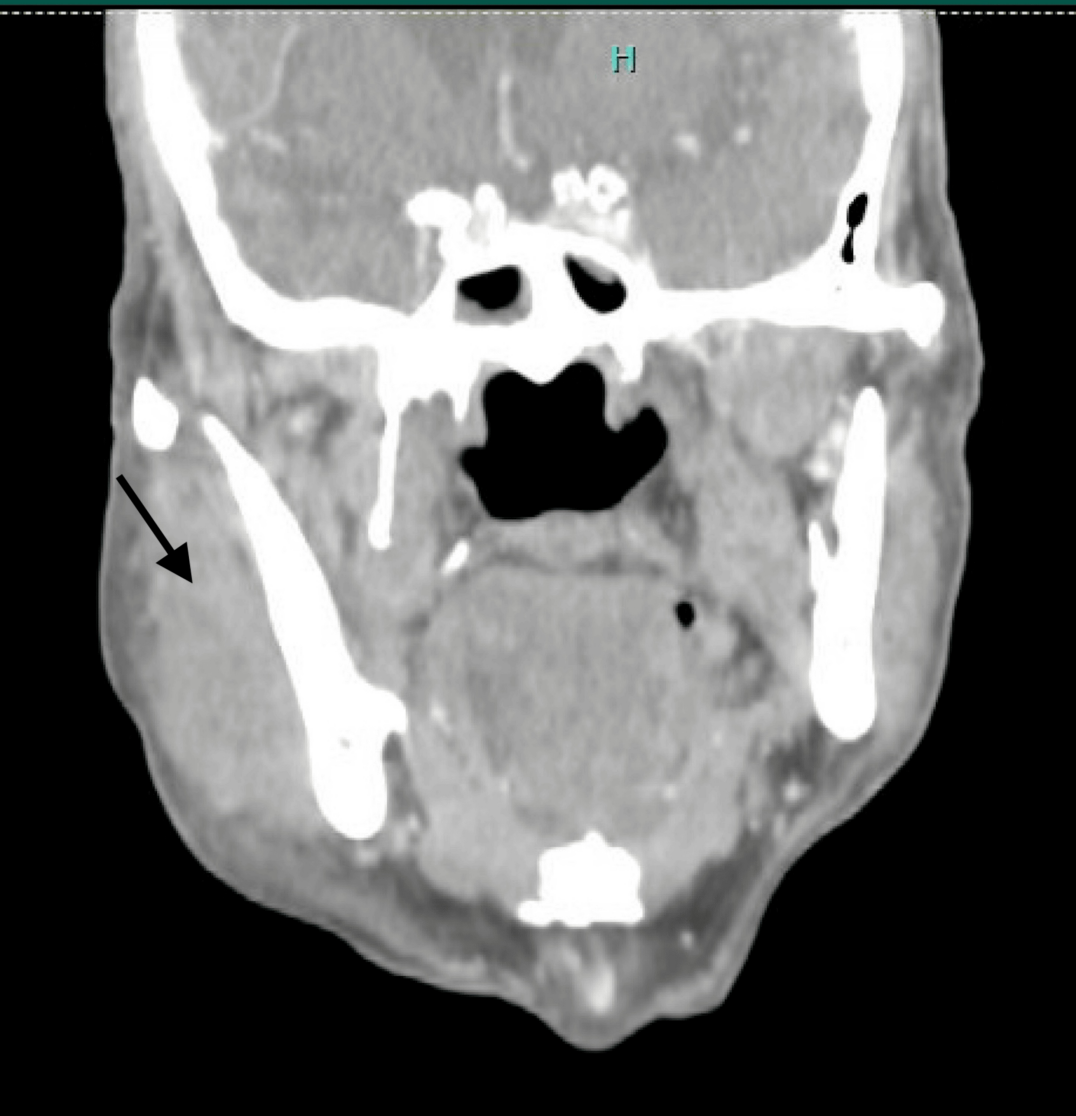
Computed tomography of the soft tissue neck demonstrating a right masseter hematoma (black arrow).

A 70-year-old African American man with a history of non-ischemic dilated cardiomyopathy (ejection fraction 40-45%), type 2 diabetes mellitus, hypertension, and former tobacco use presented to the emergency department in October 2023 with progressive dyspnea and severe anemia (hemoglobin 5.3 g/dL). He reported a one-month history of migratory subcutaneous "knots" involving the hips, lower extremities, and hands, accompanied by 20 pounds of unintentional weight loss and upper respiratory symptoms, including cough and nasal congestion. On admission, polymerase chain reaction (PCR) testing was positive for SARS-CoV-2. Notably, the patient had never received a COVID-19 vaccination. Evaluation for occult malignancy, prompted by the significant weight loss and anemia, including esophagogastroduodenoscopy, colonoscopy, and computed tomography (CT) of the chest, abdomen, and pelvis, was unremarkable. Review of the patient's medications, which included lisinopril, carvedilol, furosemide, and metformin, identified no agents with known associations with acquired hemophilia A (AHA). Antinuclear antibody screening was negative; however, rheumatoid factor and anti-cyclic citrullinated peptide antibodies were not evaluated, although the clinical presentation did not suggest underlying inflammatory arthritis or connective tissue disease. Physical examination revealed right upper extremity edema and multiple spontaneous ecchymoses without antecedent trauma. Laboratory evaluation demonstrated a severely prolonged activated partial thromboplastin time (aPTT) of 128.9 seconds with a normal prothrombin time (PT 13.0 seconds; international normalized ratio 1.2). Mixing studies revealed a partial correction to 83.9 seconds, consistent with the presence of an inhibitor. Factor VIII activity was undetectable (<1%), and the factor VIII inhibitor titer was markedly elevated at 561 Bethesda units (BU) (Table 1). CT imaging demonstrated intramuscular hematomas involving the right chest wall and right upper extremity with extensive subcutaneous edema (Figure 1). Over the ensuing days, the patient developed an expanding right masseter hematoma resulting in facial asymmetry (Figure 2). Given the temporal relationship between his prodromal upper respiratory illness and symptom onset, the absence of underlying malignancy, the lack of offending medications, and documented acute SARS-CoV-2 infection in an unvaccinated individual, COVID-19 was identified as the most likely etiologic trigger for AHA.

**Table 1:** Comparison of pertinent laboratory values with reference ranges across the disease course. PTT: partial thromboplastin time; PT: prothrombin time; INR: international normalized ratio; BU: Bethesda units; WBC: white blood cell count; ANC: absolute neutrophil count; BUN: blood urea nitrogen; AST: aspartate aminotransferase; ALT: alanine aminotransferase; LDH: lactate dehydrogenase; ANA: antinuclear antibody; ESR: erythrocyte sedimentation rate; CRP: C-reactive protein; CMV: cytomegalovirus; PCR: polymerase chain reaction; N/A: not available.

Parameter	Reference Range	Initial (Oct 2023)	Peak Abnormal
Coagulation studies			
PTT	25.1-36.5 sec	128.9 sec	128.9 sec
PT	10.2-12.9 sec	13.0 sec	74.2 sec
INR	0.9-1.1	1.2	6.8
Factor VIII activity	50-150%	<1%	<1%
Factor VIII inhibitor (Bethesda)	0 BU	561 BU	677 BU
Fibrinogen	200-400 mg/dL	520 mg/dL	520 mg/dL
Hematology			
Hemoglobin	13.0-18.0 g/dL	5.3 g/dL	4.1 g/dL
Hematocrit	35-47%	16.2%	12.3%
WBC	4.5-11.0 k/µL	5.2 k/µL	0.19 k/µL
ANC	>1.5 k/µL	2.84 k/µL	0.12 k/µL
Platelets	150-440 k/µL	163 k/µL	80 k/µL
Chemistry			
Creatinine	0.7-1.3 mg/dL	1.10 mg/dL	3.48 mg/dL
BUN	7-20 mg/dL	17 mg/dL	43 mg/dL
Potassium	3.5-5.0 mEq/L	4.5 mEq/L	7.2 mEq/L
Lactate	<2.0 mmol/L	N/A	19.6 mmol/L
AST	10-40 U/L	Normal	>1,000 U/L
ALT	7-56 U/L	Normal	>400 U/L
Total bilirubin	0.1-1.2 mg/dL	Normal	2.3 mg/dL
LDH	140-280 U/L	433 U/L	3,859 U/L
Albumin	3.5-5.0 g/dL	2.8 g/dL	1.8 g/dL
Infectious/inflammatory			
COVID-19 PCR	Negative	Positive	Positive
ANA	Negative	Negative	N/A
ESR	0-22 mm/hr	15 mm/hr	63 mm/hr
CRP	<3.0 mg/L	73.4 mg/L	142.3 mg/L
Procalcitonin	<0.5 ng/mL	N/A	747.64 ng/mL
Beta-D-glucan	<60 pg/mL	N/A	158 pg/mL
CMV viral load	Not detected	N/A	298 IU/mL
C. difficile	Negative	Negative	Positive

Treatment was initiated with activated prothrombin complex concentrate (FEIBA) 100 units/kg every 12 hours for bypassing therapy, prednisone 1 mg/kg daily, and cyclophosphamide 100 mg daily for immunosuppression. Despite initial therapy, the inhibitor titer paradoxically rose to a peak of 677 BU before beginning to decline, prompting the addition of rituximab 375 mg/m² weekly. By late October, the hematomas were resolving and hemoglobin had stabilized, permitting discharge on prednisone and cyclophosphamide with scheduled outpatient rituximab infusions. Subsequent follow-up demonstrated a gradual decline in inhibitor titer from 677 BU to 208 BU, then to 88 BU by early December.

**Figure 3:**
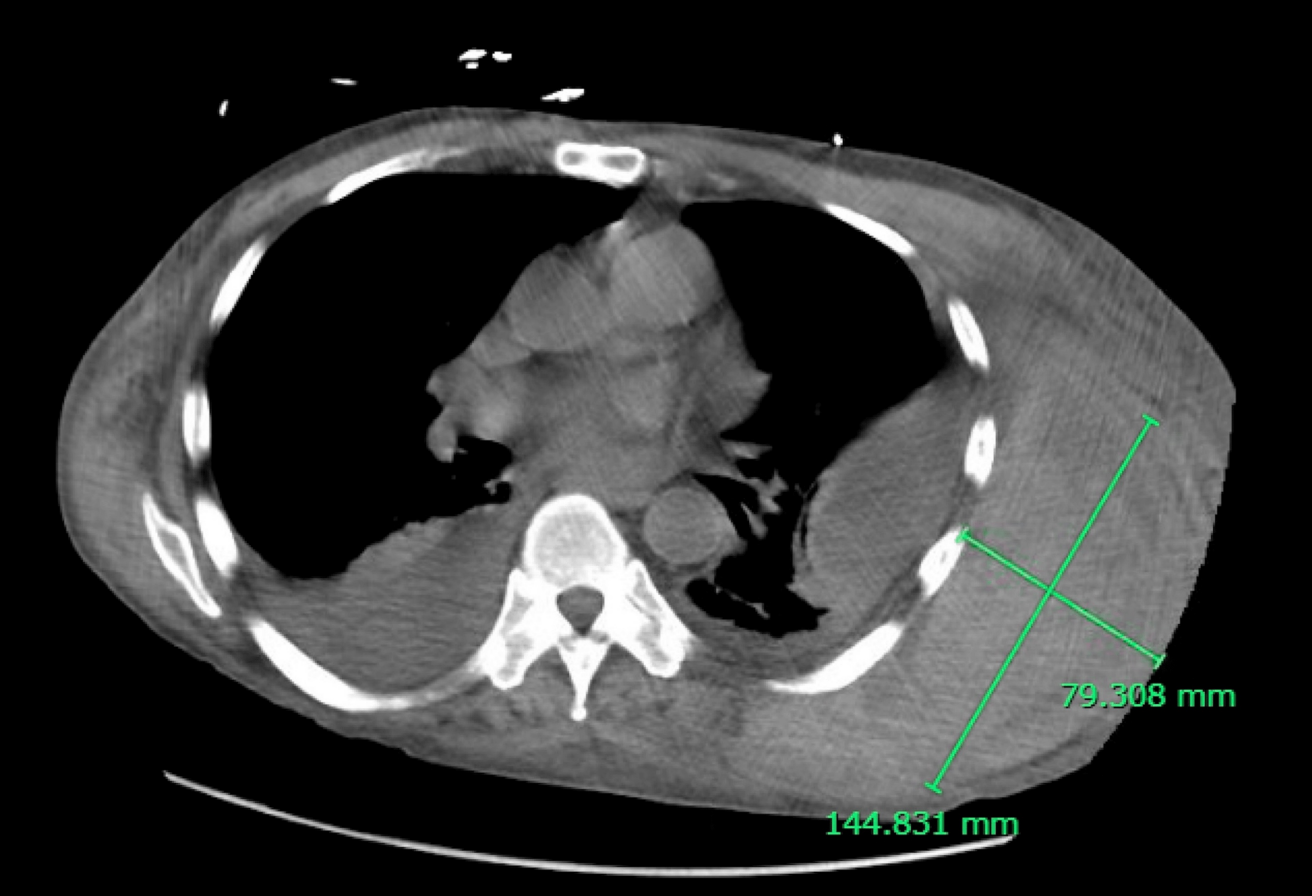
Axial computed tomography of the chest demonstrating a large left chest wall hematoma.

In early December 2023, the patient was found unresponsive at home and subsequently developed pulseless electrical activity (PEA) cardiac arrest, with return of spontaneous circulation achieved after approximately four minutes of cardiopulmonary resuscitation. Post-arrest CT of the chest revealed a massive left posterolateral chest wall hematoma (Figure 3). The clinical course was complicated by hemorrhagic shock and multisystem organ dysfunction. Management required vasopressor support, activation of the massive transfusion protocol, and mechanical ventilation. FEIBA was reinitiated, and high-dose intravenous methylprednisolone was administered. The hospitalization was further complicated by vancomycin-induced agranulocytosis (absolute neutrophil count nadir 0.12 K/μL), cytomegalovirus reactivation requiring antiviral therapy, and Clostridioides difficile colitis. Despite these setbacks, the patient gradually improved, was successfully extubated after four days, and completed his four-dose rituximab course. He was discharged to inpatient rehabilitation in late December 2023 and returned home in early January 2024.

At outpatient follow-up in mid-January 2024, laboratory studies confirmed complete immunological remission with factor VIII activity of 343% and a Bethesda inhibitor titer of 0 BU. However, in late January 2024, the patient presented to the hospital with progressive dyspnea, hypoxia, and atrial fibrillation with rapid ventricular response. He rapidly decompensated, requiring endotracheal intubation and escalating vasopressor support. Laboratory evaluation revealed profound pancytopenia (white blood cell count 0.19 K/μL), acute kidney injury, hyperkalemia, and severe lactic acidosis. Procalcitonin was critically elevated at 747.64 ng/mL, and beta-D-glucan was positive (158 pg/mL), indicating overwhelming bacterial sepsis with possible invasive fungal co-infection (Table 1). Despite aggressive supportive care, including continuous renal replacement therapy and broad-spectrum antimicrobial and antifungal therapy, multiorgan failure progressed inexorably. Following goals-of-care discussions with the family, comfort-focused measures were initiated. The patient died in late January 2024, approximately three months after his initial AHA diagnosis, with his family at bedside. The cause of death was documented as septic shock secondary to profound immunosuppression.

## Discussion

This case represents one of the highest reported factor VIII inhibitor titers in COVID-19-associated AHA. While the European Acquired Haemophilia Registry (EACH2) reported a median inhibitor titer of 19 BU in 501 patients with AHA from all etiologies [9], our patient presented with a titer of 561 BU that peaked at 677 BU, approximately 35-fold higher than the registry median. In the specific context of COVID-19-associated AHA, previously reported titers have ranged from 8.49 to 318 BU [7,10-11], making this case exceptional in its severity. The association between COVID-19 and AHA adds to the growing literature on immune dysregulation following SARS-CoV-2 infection, with proposed mechanisms including molecular mimicry between viral antigens and factor VIII epitopes, as well as dysregulated T and B cell activation in the context of COVID-19-associated cytokine storm [5,6]. Notably, our patient had documented PCR-positive COVID-19 infection as a clear temporal trigger, without the confounding factor of recent vaccination seen in some reported cases.

Despite the extraordinarily high inhibitor titer, our patient achieved complete remission (factor VIII 343%, inhibitor 0 BU) with a regimen of corticosteroids, cyclophosphamide, and rituximab, consistent with international guidelines recommending combination therapy for high-titer or refractory disease [1,8]. The addition of rituximab appears to have been critical, as the inhibitor titer initially increased on steroids and cyclophosphamide alone before declining after rituximab initiation. However, this case illustrates the therapeutic paradox inherent to AHA treatment: the same intensive immunosuppression required to eradicate the inhibitor created profound susceptibility to infection. Our patient developed multiple infectious complications, including CMV reactivation, Clostridioides difficile colitis, and ultimately overwhelming sepsis (procalcitonin 747.64 ng/mL) with possible invasive fungal infection (positive beta-D-glucan). This outcome aligns with the EACH2 registry data reporting that infections related to immunosuppression were the leading cause of death in AHA patients who achieved remission [8].

The emergence of emicizumab, a bispecific antibody mimicking factor VIII cofactor function, offers a potential paradigm shift in AHA management that may mitigate this therapeutic paradox. A recent systematic review of 171 AHA patients treated with emicizumab demonstrated effective bleeding control with a favorable safety profile [12]. Notably, the GTH-AHA-EMI trial showed that emicizumab prophylaxis without concurrent immunosuppressive therapy during the initial 12 weeks reduced both bleeding rates and fatal infections compared with historical controls receiving immunosuppression alone, with 24-week survival rates of 90% versus 76% [13,14]. For patients like ours with exceptionally high inhibitor titers requiring prolonged intensive immunosuppression, emicizumab could theoretically provide hemostatic protection while allowing delayed or reduced-intensity immunosuppressive regimens, potentially avoiding the fatal infectious complications that ultimately claimed our patient’s life. However, significant barriers limit emicizumab accessibility in AHA. The drug remains off-label for AHA in most countries outside Japan, and the cost, approximately $500,000 annually at standard dosing, presents a substantial obstacle, particularly for uninsured or underinsured patients [15]. This economic reality may disproportionately affect patients from underserved communities, compounding existing healthcare disparities.

Finally, this case highlights important disparities in AHA recognition and representation. Our patient, an African American male, represents a demographic potentially underrepresented in AHA literature, which predominantly describes Caucasian populations [2]. The cutaneous manifestations of AHA, ecchymoses and subcutaneous hematomas, may appear differently on darker skin tones, presenting as deeper purple, brown, or hyperpigmented lesions rather than the classic red-blue discoloration typically depicted in medical literature [16]. The paucity of clinical images representing these findings across diverse skin tones has been associated with diagnostic delays and worse health outcomes in patients with skin of color [17]. As evidence supporting emerging therapies like emicizumab continues to accumulate, advocacy for expanded access, insurance coverage, and diverse representation in medical education will be essential to ensure equitable outcomes for all patients with this life-threatening condition.

## Conclusions

This case of COVID-19-associated AHA with an inhibitor titer of 561-677 BU, among the highest reported in the literature, demonstrates that complete remission is achievable even with extraordinarily high titers through combination immunosuppressive therapy. However, our patient’s death from treatment-related sepsis despite achieving remission underscores the therapeutic paradox central to AHA management. Emerging therapies such as emicizumab may help mitigate this paradox by providing hemostatic protection while allowing reduced-intensity immunosuppression, though cost and off-label status currently limit accessibility. This case highlights the ongoing need to optimize treatment strategies that balance bleeding control with infection prevention while ensuring equitable access to novel therapeutics.
